# Biomechanical analysis of the relation between movement time and joint moment development during a sit-to-stand task

**DOI:** 10.1186/1475-925X-8-27

**Published:** 2009-10-22

**Authors:** Shinsuke Yoshioka, Akinori Nagano, Dean C Hay, Senshi Fukashiro

**Affiliations:** 1 Department of Life Sciences (Sports Sciences), The University of Tokyo, Japan; 2 Department of Computer Science and Systems Engineering, Kobe University, Japan; 3 School of Physical and Health Education, Nipissing University, Ontario, Canada

## Abstract

**Background:**

Slowness of movement is a factor that may cause a decrease of quality of daily life. Mobility in the elderly and people with movement impairments may be improved by increasing the quickness of fundamental locomotor tasks. Because it has not been revealed how much muscle strength is required to improve quickness, the purpose of this study was to reveal the relation between movement time and the required muscle strength in a sit to stand (STS) task. Previous research found that the sum of the peak hip and knee joint moments was relatively invariant throughout a range of movement patterns (Yoshioka et al., 2007, Biomedical Engineering Online 6:26). The sum of the peak hip and knee joint moment is an appropriate index to evaluate the muscle strength required for an STS task, since the effect of the movement pattern variation can be reduced, that is, the results can be evaluated purely from the viewpoint of the movement times. Therefore, the sum of the peak hip and knee joint moment was used as the index to indicate the required muscle strength.

**Methods:**

Experimental kinematics data were collected from 11 subjects. The time at which the vertical position of the right shoulder fell outside three standard deviations of the vertical positions during the static initial posture was regarded as the start time. The time at which the vertical position fell within three standard deviations of the vertical positions during static upright standing posture was regarded as the finish time. Each movement time of the experimental movements was linearly lengthened and shortened through post-processing. Combining the experimental procedure and the post-processing, movements having various movement patterns and a wide range of movement times were obtained. The joint moment and the static and inertial components of the joint moment were calculated with an inverse dynamics method. The static component reflects the gravitational and/or external forces, while the inertial component reflects the acceleration of the body.

**Results:**

The quantitative relation between the movement time and the sum of the peak hip and knee joint moments were obtained. As the STS movement time increased, the joint moments decreased exponentially and converged to the static component (1.51 ~ 1.54 N.m/kg). When the movement time was the longest (movement time: 7.0 seconds), the joint moments (1.57 N.m/kg) closely corresponded to the minimum of 1.53 N.m/kg as reported by Yoshioka et al..

**Conclusion:**

The key findings of this study are as follows. (1) The minimum required joint moment for an STS task is essentially equivalent to the static component of the joint moment. (2) For fast and moderate speed movements (less than 2.5 seconds), joint moments increased exponentially as the movement speed increased. (3) For slow movements greater than 2.5 seconds, the joint moments were relatively constant. The results of this STS research has practical applications, especially in rehabilitations and exercise prescription where improved movement time is an intended target, since the required muscle strength can be quantitatively estimated.

## Background

A sit-to-stand (STS) movement, which is defined as a movement of standing up from a chair to an upright posture, is one of the most demanding daily activities in mechanical terms. An STS movement requires a peak joint moment greater than other movements such as stair climbing or walking [1], and yields peak hip joint contact pressure higher than other movements such as walking, jogging or jumping [2]. Hodge et al. [2] used a specially built hip endoprosthesis equipped with pressure measuring transducers. They showed that the peak hip contact pressure between the acetabulum of the pelvis and the femoral head during an STS movement was greater than that during walking, jogging or jumping. Also, an STS movement requires muscle strength greater than other daily activities, such as walking or stair climbing [3]. Because of these mechanical demands, there are many elderly people who experience difficulty when standing up from a chair [4,5]. Such difficulties influence their quality of daily life and the ability to remain independent.

Slowness of movement may be a factor causing a decrease of quality of daily life. The STS movement times of young people are generally less than 2 seconds [6,7]. On the other hand, those of the elderly or people with mobility impairments are more than 2 seconds [4,8,9]. Mobility in these at-risk populations may be improved by increasing STS quickness. However, it has not been revealed how much muscle strength is required to improve the quickness of this fundamental movement task. Therefore, the purpose of this study was to reveal the relation between movement time and the required muscle strength.

Yoshioka et al. [10] focused on the minimum muscle strength required for an STS task by combining experimental and simulated data of 160,086 STS movements. They calculated the joint moments during all movements and revealed that the sum of the peak hip and knee joint moments needed to be greater than 1.53 N.m/kg in order to achieve an STS task. While the individual peak joint moments were greatly affected by the movement patterns, the sum of the peak hip and knee joint moments was relatively invariant throughout the range of movement patterns. The sum of the peak hip and knee joint moment is an appropriate index to evaluate the muscle strength required for an STS task, since the effect of the movement pattern can be removed, that is, the results can be evaluated purely from the view point of the movement times. Therefore, the sum of the peak hip and knee joint moment was used as the index to indicate the required muscle strength in the current study.

## Methods

Eleven healthy young male subjects (age 25 ± 2 years, height 1.72 ± 0.04 m, mass 70.9 ± 4.8 kg) participated in this experiment with informed consent. None of them had any known musculoskeletal or neurological disorders. This project was performed under the approval of the ethics committee of the University of Tokyo.

Each subject was instructed to perform a total of fifteen STS movements using self-selected speeds and movement patterns without arm support. The initial posture and feet position of the subjects were not restricted. A brief rest time was assigned between trials. The first five trials were treated as practice, though the subjects were not informed of this. Therefore, 110 trials (10 trials per subject × 11 subjects) were used for analysis. Based on the Japan Industrial Standard (JIS S 1011 and JIS S 1015) and British Standards Institute [11], chair height was set to 0.40 m.

To obtain the kinematics, three-dimensional coordinates of the landmark points of the subject's body were acquired using a 3D optical motion capture system with 7 cameras at 200 Hz (Hawk Digital System, Motion Analysis Corporation, Santa Rosa, CA, USA). Seven reflective markers were placed on the subject's body (the right acromion, sacroiliac joint, right and left anterior superior iliac spines, right epicondylus lateralis, right malleolus lateralis and the distal end of the fifth metatarsal). All raw coordinates data were smoothed using a fourth-order Butterworth lowpass digital filter. The cutoff frequency (lower than 10 Hz) was determined with a residual analysis [12]. The hip joint center position was calculated from the sacroiliac joint, right and left anterior superior iliac spines and right epicondylus lateralis [13].

The STS start and finish time was determined with the marker attached on the right acromion. The time at which the vertical marker position fell outside three standard deviations of the vertical marker positions during the static initial posture was regarded as the start time. The time at which the vertical marker position fell within three standard deviations of the vertical marker positions during static upright standing posture was regarded as the finish time. The load imposed on the chair seat was measured with a force platform (9281B, Kistler Instrumente AG, Winterthur, Switzerland) placed under the chair. The time at which the load fell below 10 N was regarded as the seat off time.

**Figure 1:**
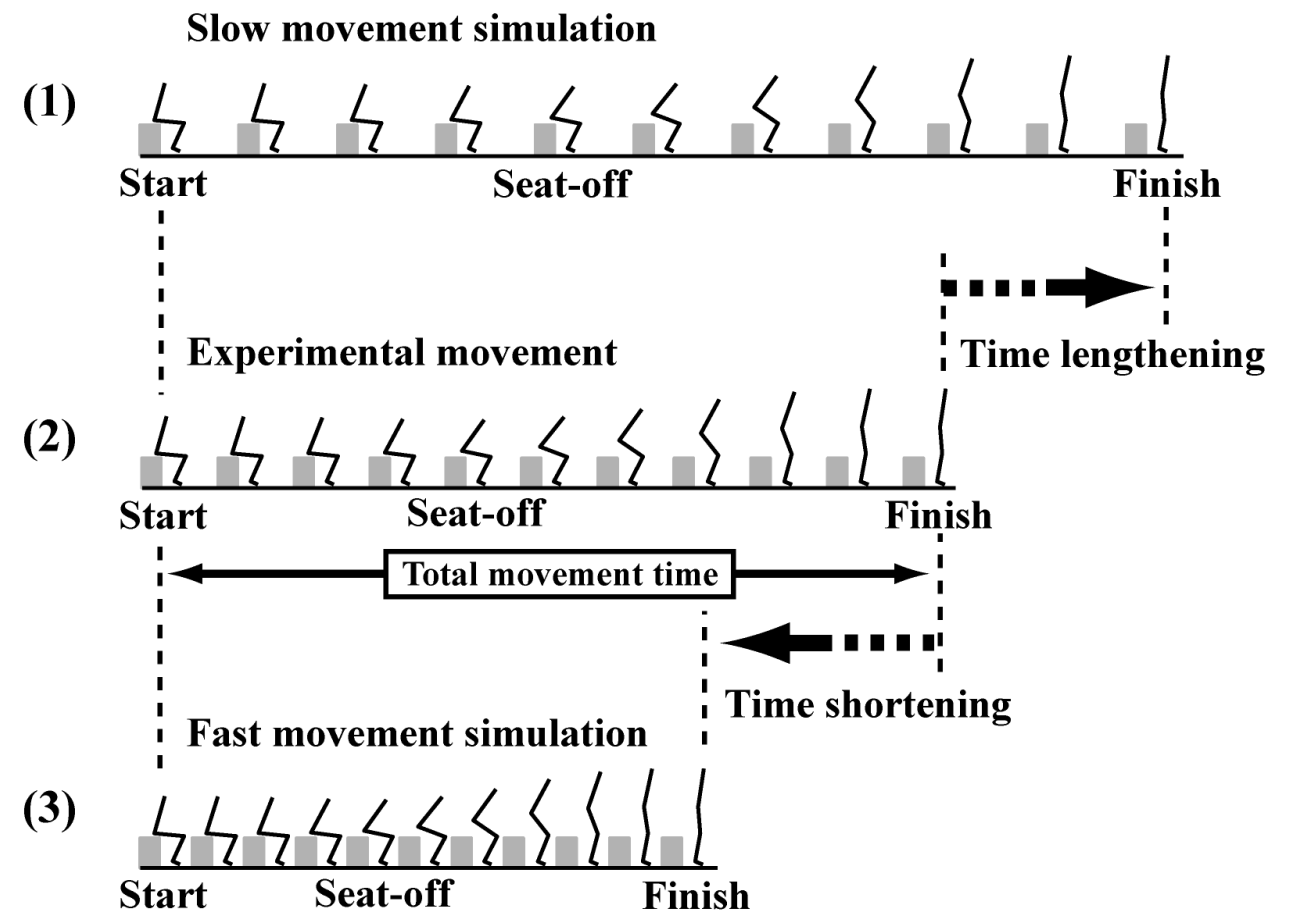
**The process to obtain movements over a wide range of movement times**. The movements of various durations were obtained by linearly lengthening or shortening the movement time of the experimental kinematics data. The stick pictures of the slow movement (1) and the fast movement (3) are the example pictures obtained by lengthening and shortening the movement time of the experimental kinematics data (2), respectively. Through this process, from each experimental trial, sixty-one STS movements having the same movement pattern but different movement times were obtained ranging from 1.0 to 7.0 seconds in 0.1 seconds steps.

The STS movement times reported in previous studies range from 1 to 6 seconds. It is necessary to investigate a wide range of STS movements, but it is not easy to obtain movements of durations longer than 6 seconds using only experimental procedures as most subjects do not normally perform such slow movements. Therefore, in this study, by linearly lengthening or shortening the movement time of each experimental trial, movements of various durations were obtained through simulation (Fig. 1). Through this process, from each experimental trial, sixty-one STS movements having the same movement pattern but different movement times were obtained ranging from 1.0 to 7.0 seconds in 0.1 seconds steps. Combining the experimental procedure and this post-processing, movements having various movement patterns and wide ranging movement times could be obtained. The appropriateness of this method should be considered, since there are reports stating that the movement patterns may change with increased movement speed [14,15]. The use of the method in the current study was assumed to be adequate for the following reasons. First, any changes in movement patterns due to movement speed are more likely to be subject-specific, and not indicative of a general trend. This is supported by previous research that reported that movement patterns did not depend on the movement speed [16]. Second, the effect of the change of the movement pattern on the sum of the peak hip and knee joint moments is estimated to be small, since it has been reported that the sum was a relatively invariant index throughout a wide range of movement patterns [10].

The joint moments (hip, knee and ankle joints) and the coordinates of the center of pressure (COP) on the floor were calculated using an inverse dynamics method. A joint moment can be divided into two components, i.e., static and inertial components [17,18]. The static component reflects the gravitational or external forces, while the inertial component reflects the acceleration of the body. In this study, the joint moments were divided into two components (static and inertial components) according to the definition of Wu and Ladin [18] (Appendix). Inverse dynamic calculation was applied from the HAT segment toward the foot segment with the motion data and the human body segmental parameters reported in previous studies [12,19]. The details about the calculation of the joint moment are described in the Appendix section Throughout this study, bilateral symmetry was assumed, and two-dimensional analyses on the sagittal plane were applied. In this study, joint moment development during the movement was the focus of analysis. In the sitting phase, the load imposed on the lower limb is small, since the body is supported by the chair. Additionally, Schenkman et al., Kotake et al. and Kralj et al. reported that the joint moments reached the maximum after the buttocks lose contact with the chair [20-22]. Therefore, only the rising phase of the STS movement was analyzed.

The movements in which the coordinates of the COP did not stay within the subject's foot support range were assumed to be unsuccessful, and were excluded from further analysis. Joint moments of hip extension, knee extension and ankle plantar flexion were defined as positive. According to the model's dynamic equations, the values of joint moment changes linearly with the model's mass. Therefore, the joint moments were normalized by the mass of the whole body.

## Results

One hundred ten STS movements were obtained through human trials. The average movement time (standard deviation) was 1.32 (0.33) seconds. The minimum and maximum movement time was 0.79 and 2.06 seconds, respectively. The average hip joint height at the initial sitting posture was 0.492 m.

**Figure 2:**
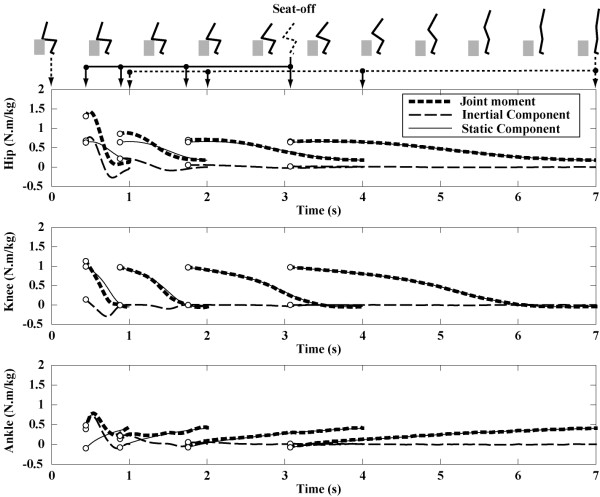
**Joint moment profiles during four STS movements**. The joint moments and the inertial and static components of the joint moment during four representative movements (movement times: 1.0, 2.0, 4.0 and 7.0 seconds) are shown. Zero seconds is the start time. The solid arrows below the stick figures indicate when seat off occurred (0.44, 0.88, 1.76 and 3.07 seconds) for each of the movements. Dashed arrows indicate when the standing posture was achieved. Joint moments during the sitting phase are not shown, since only the rising phase was analyzed. Circle plots indicate the results at the seat-off time. In the case of the slow movements (movement time: 4.0 and 7.0 seconds), the inertial components of joint moments were always nearly zero. In other words, the total of joint moment closely corresponded to the static component of the joint moment.

Sixty-one simulated movements (1.0 to 7.0 seconds, 0.1 seconds step) were obtained from each experimental trial, resulting in 6710 (110 × 61) total simulated STS movements. Fig. 2 shows an example of the results of the joint moment and the inertial and static components of the joint moment during four representative movements (movement times: 1.0, 2.0, 4.0 and 7.0 seconds) of the sixty-one simulated movements. Joint moments during the sitting phase were not shown, since only the rising phase was analyzed. Zero seconds is the start time. Circle plots indicate the results at the seat-off time. The seat-off times were 0.44, 0.88, 1.76 and 3.07 seconds, respectively. In the case of slow movements (movement times: 4.0 and 7.0 seconds), the inertial components were always nearly zero. In other words, the joint moment closely corresponded to the static component.

**Figure 3:**
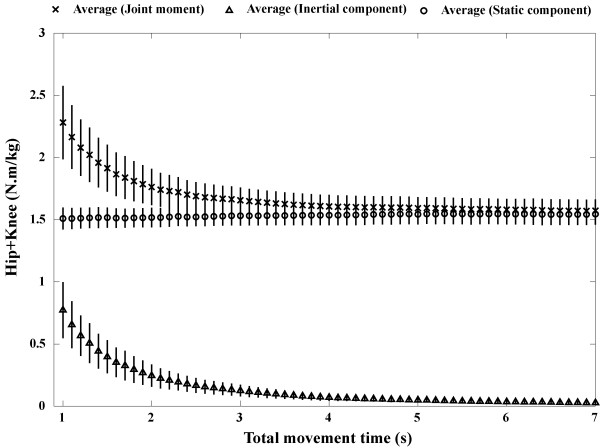
**The relation between total movement times and the sum of the peak hip and knee joint moments**. The cross, triangular and circle marks show the averages of the sum of the peak hip and knee joint moments, the inertial components and the static components, respectively (standard deviation bars are also shown). As the movement time decreased, the sum of those moments and the inertial components increased. On the other hand, the static components were relatively constant. This figure is useful to estimate the required joint moment for each movement time.

The average and standard deviation of the sum of the peak hip and knee joint moments at each movement time are shown in Fig. 3. The average and standard deviation of the peak values of the inertial and static components are also shown in Fig. 3. (The horizontal axes of Fig. 3 show the total movement times, and those of Fig. 2 show the time sequence. The horizontal axes of Fig. 2 are different from those of Fig. 3.) As the STS movement times increased, the sum of the peak hip and knee joint moments and the inertial component decreased. On the other hand, the static component was relatively constant. As the STS movement times increased, the sum of the peak hip and knee joint moments converged on the static component, since the inertial component converged on zero value. At the slowest movement (7.0 seconds), the inertial component was nearly zero (0.03 N.m/kg).

## Discussion

The purpose of this study was to reveal the relation between the movement time and the muscle strength required for an STS task (the sum of the peak hip and knee joint moments during a STS task).

The quantitative relation between the movement time and the sum of those moments was revealed (Fig. 3). As the movement time decreased, the sum of the peak hip and knee joint moments increased. In particular, when the movement times were relatively short, the required joint moments increased exponentially. Using this finding, the amount of the required joint moment for each movement time can be estimated. For example, to successfully stand in 1.5 seconds, about 1.8 N.m/kg is required. This finding is useful for practical applications where the time of movement is important.

The static and inertial components were separately calculated. It was revealed that the static components (1.51 ~ 1.54 N.m/kg) closely correspond to the minimum required joint moment to achieve an STS task (1.53 N.m/kg) [10]. From Fig. 3, it can be estimated that the inertial components during the slow movements (4.12 - 10.98 seconds) in Yoshioka et al. were nearly zero. That is to say, in mechanical terms, it can be stated that the minimum required joint moment is essentially equivalent to the static component of the joint moment. This is an interesting and important finding. This finding suggests that, by examining the static component, it is possible to reveal the determinant of the minimum required joint moment. For example, how a chair height, arm support or body height affect the minimum required joint moment? These are recognized to be themes of future work.

**Figure 4:**
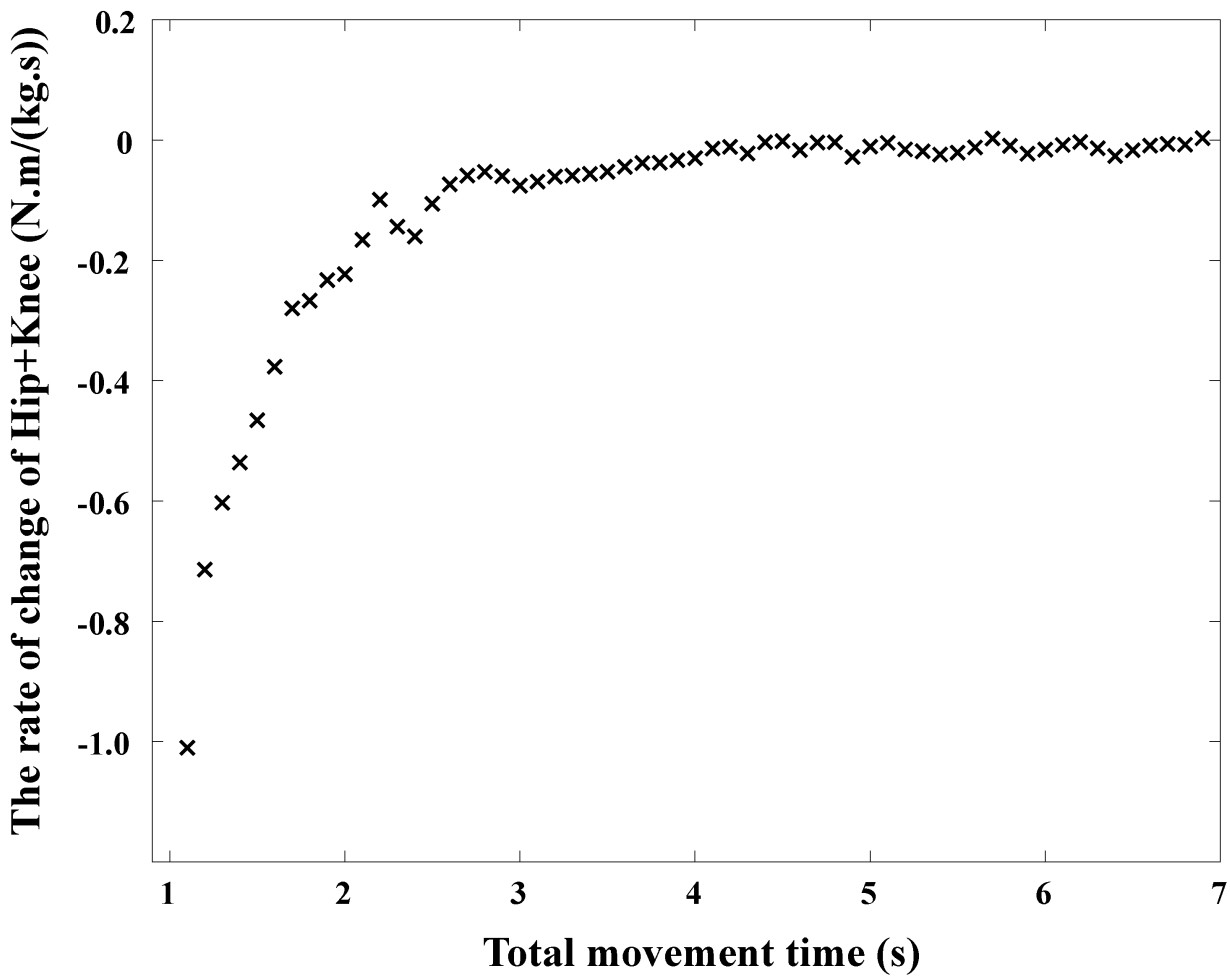
**The relation between total movement times and the rate of change of the sum of the peak hip and knee joint moments**. When the movement times were below 2 ~ 3 seconds, the values changed exponentially. On the other hand, when the movement times were above 2 ~ 3 seconds, the values were relatively constant.

Fig. 4 shows the rate of change of the sum of the peak hip and knee joint moments. It was revealed that, when the movement times were below 2.5 seconds, as the movement time decreased, the sum of those moments increased exponentially. On the other hand, when the movement times were above 2.5 seconds, the sum of those moments was relatively constant. These results suggest that a 2 ~ 3 seconds movement may be efficient, since it is rapid enough that fatigue does not occur, but slow enough to reduce peak joint moments. Published reports of STS movement times ranged from 1.2 to 5.9 seconds, while the most frequent time within those studies is about 2 seconds. It might be said that most people choose 2 seconds to perform an STS movement unconsciously because it is an efficient duration.

It can be said that, when the movement time is above 2.5 seconds, the mechanical load of an STS task is relatively constant. That is to say, this finding suggests that if the people have at least the minimal physical strength and coordination to stand up once, they can stand up in about 2.5 seconds. However, there are people who take more than 2.5 seconds to stand up. Schultz et al. revealed that balance ability is important for an STS task [23]. The balance ability of people who required more than 2.5 seconds to stand may be low. Nevitt et al. reported that, for people who could not stand up or took more than 2 seconds to achieve an STS task, the risk of two or more falls in one year was 2.4 times the risk of one or no fall [24]. Both studies suggest a link between movement time, balance and risk of falling, with strength being an implicit factor. It will require further study to fully understand these complex interactions.

It has been reported that the movement strategy of obese people was different from that of non-obese [25]. The joint moment profiles of the hip and knee joints were different between the two groups, since the obese people stood up from a chair with a more upright trunk position. In the case of obese people, the hip joint moments were smaller than the knee joint moments. On the other hand, in the case of non-obese people, the hip joint moments were greater than the knee joint moments. It is necessary to discuss the effect of the difference of the movement strategy on the results of this study. Yoshioka et al. reported that the sum of peak hip joint moment and peak knee joint moment was relatively invariant throughout the range of movement strategies, although each of the peak joint moments was greatly affected by the movement strategy [10]. In Sibella et al. [25], similar trend was observed. Each of the peak joint moments was different between obese people (Hip 0.57 N.m/kg, Knee 0.77 N.m/kg) and non-obese people (Hip 0.88 N.m/kg, Knee 0.45 N.m/kg). However, the sum of peak hip and knee joint moment of obese people (1.34 N.m/kg) was closely similar to that of non-obese people (1.33 N.m/kg). From these results, it is suggested that the findings of this study can be applied not only to healthy, normal body weight people, but also to obese people.

In this study, to focus on the hip and knee joint moments, the upper limb use was not taken into consideration. Empirically, it has been recognized that using the upper limb can make the STS movement easier. Actually, Seedhom & Terayama and Ellis et al. revealed that the use of upper limb reduced the mechanical load of the knee joint [26,27]. O'Meara & Smith further developed the findings of the previous studies [28,29]. They examined the grab rail assistance and found that, although each lower limb joint (including knee joint) was affected by the assistance, the overall lower limb effort was not affected. This finding suggests that the effect of the upper limb use on the sum of the peak hip and knee joint moments is small. That is to say, the effect of the upper limb use on the results of this study is small. However, as possible effects have not been examined, we acknowledge that future studies should consider upper limb use.

As the movement time decreased, the required muscle strength to achieve an STS task increased, since the inertia increased. Using this mechanism, some kinds of STS tests have been developed as a field test to evaluate the lower leg strength conveniently [30-32] and have been widely utilized [33]. From the results of STS tests [31,34,35] and the time ratio between the sit-to-stand and stand-to-sit movements (1:1) [36], it can be estimated that the STS times of the low physical capacity group were approximately 2 ~ 3 seconds. There were few people who took more than 3 or 4 seconds to achieve an STS task. This result would support the finding of this study that the mechanical load of an STS task is relatively constant, when the movement time is above 2.5 seconds.

## Conclusion

The purpose of this study was to reveal the quantitative relation between movement time and the sum of the peak hip and knee joint moments during a sit-to-stand task. The key findings of this study are as follows. (1) The minimum required joint moment for an STS task was essentially equivalent to the static component of the joint moment. (2) For fast and moderate speed movements (less than 2.5 seconds), as the movement speed increased, the joint moments increased exponentially. (3) For slow movements (more than 2.5 seconds), the joint moments were relatively constant. The results of this STS research has practical applications, especially in rehabilitations and exercise prescription where improved movement time is an intended target, since the required muscle strength can be quantitatively estimated.

## Competing interests

The authors declare that they have no competing interests.

## Authors' contributions

SY performed the data collection and analyses, constructed the simulation model and drafted the manuscript. AN participated in the process of data collection, analysis, model construction and manuscript writing. DCH and SF contributed discussions and suggestions throughout this project, including the manuscript writing. All authors read and approved the final manuscript.

## Appendix

1) Equations of the joint moments and the inertial and static components of the joint moment

The following equations were used to calculate the joint moments and the inertial and static components of the joint moment. In this study, bilateral symmetry and two dimensional motion on the sagittal plane were assumed. Therefore, a half of the mass and moment of inertia was considered for each segment.

(Hip joint moment)

(1)

(2)

(3)

(Knee joint moment)

(4)

(5)

(6)

(Ankle joint moment)

(7)

(8)

(9)

2) Nomenclature

HAT head-arm-trunk segment

THIGH thigh segment

SHANK shank segment

HIP hip joint

KNEE knee joint

ANKLE ankle joint

 joint moment vector about joint j

Joint moments of hip extension, knee extension and ankle plantar flexion were defined as positive.

 inertial component vector of the joint moment about joint j

 static component vector of the joint moment about joint j

*I*_*i *_moment of inertia of segment *i *about the center of mass

 angular acceleration vector of segment *i *about the center of mass

 position vector from joint j to the center of gravity of segment I

*body_mass *mass of whole body

*m*_*i *_mass of segment *I*

 acceleration vector of the center of gravity of segment i

 acceleration vector of gravity

## References

[B1] Rodosky MW, Andriacchi TP, Andersson GBJ (1989). The Influence Of Chair Height On Lower-Limb Mechanics During Rising. Journal of Orthopaedic Research.

[B2] Hodge WA, Carlson KL, Fijan RS, Burgess RG, Riley PO, Harris WH, Mann RW (1989). Contact Pressures From An Instrumented Hip Endoprosthesis. Journal of Bone and Joint Surgery American Volume.

[B3] Ploutz-Snyder LL, Manini T, Ploutz-Snyder RJ, Wolf DA (2002). Functionally relevant thresholds of quadriceps femoris strength. Journals of Gerontology Series A, Biological Sciences and Medical Sciences.

[B4] Alexander NB, Schultz AB, Warwick DN (1991). Rising From A Chair - Effects Of Age And Functional Ability On Performance Biomechanics. Journals of Gerontology.

[B5] Schultz AB (1995). Muscle function and mobility biomechanics in the elderly: an overview of some recent research. Journals of Gerontology Series A, Biological Sciences and Medical Sciences.

[B6] Papa E, Cappozzo A (2000). Sit-to-stand motor strategies investigated in able-bodied young and elderly subjects. Journal of Biomechanics.

[B7] Shepherd RB, Koh HP (1996). Some biomechanical consequences of varying foot placement in sit-to-stand in young women. Scandinavian Journal of Rehabilitation Medicine.

[B8] Engardt M, Olsson E (1992). Body weight-bearing while rising and sitting down in patients with stroke. Scandinavian Journal of Rehabilitation Medicine.

[B9] Galli M, Cimolin V, Crivellini M, Campanini I (2008). Quantitative analysis of sit to stand movement: experimental set-up definition and application to healthy and hemiplegic adults. Gait and Posture.

[B10] Yoshioka S, Nagano A, Himeno R, Fukashiro S (2007). Computation of the kinematics and the minimum peak joint moments of sit-to-stand movements. Biomedical Engineering Online.

[B11] Skelton DA, Greig CA, Davies JM, Young A (1994). Strength, power and related functional ability of healthy people aged 65-89 years. Age Ageing.

[B12] Winter DA (1990). Biomechanics and Motor Control of Human Movement.

[B13] Besier TF, Sturnieks DL, Alderson JA, Lloyd DG (2003). Repeatability of gait data using a functional hip joint centre and a mean helical knee axis. Journal of Biomechanics.

[B14] Pai YC, Rogers MW (1991). Speed Variation And Resultant Joint Torques During Sit-To-Stand. Archives of Physical Medicine and Rehabilitation.

[B15] Gross MM, Stevenson PJ, Charette SL, Pyka G, Marcus R (1998). Effect of muscle strength and movement speed on the biomechanics of rising from a chair in healthy elderly and young women. Gait and Posture.

[B16] Linden DWV, Brunt D, McCulloch MU (1994). Variant and Invariant Characteristics of the Sit-to-Stand Task in Healthy Elderly Adults. Archives of Physical Medicine and Rehabilitation.

[B17] Hof AL (1992). An explicit expression for the moment in multibody systems. Journal of Biomechanics.

[B18] Wu G, Ladin Z (1996). Limitations of quasi-static estimation of human joint loading during locomotion. Medical & Biological Engineering & Computing.

[B19] de Leva P (1996). Adjustments to Zatsiorsky-Seluyanov's segment inertia parameters. Journal of Biomechanics.

[B20] Kotake T, Dohi N, Kajiwara T, Sumi N, Koyama Y, Miura T (1993). An Analysis Of Sit-To-Stand Movements. Archives of Physical Medicine and Rehabilitation.

[B21] Kralj A, Jaeger RJ, Munih M (1990). Analysis of standing up and sitting down in humans: definitions and normative data presentation. Journal of Biomechanics.

[B22] Schenkman M, Berger RA, Riley PO, Mann RW, Hodge WA (1990). Whole-Body Movements During Rising To Standing From Sitting. Physical Therapy.

[B23] Schultz AB, Alexander NB, Ashtonmiller JA (1992). Biomechanical Analyses Of Rising From A Chair. Journal of Biomechanics.

[B24] Nevitt MC, Cummings SR, Kidd S, Black D (1989). Risk factors for recurrent nonsyncopal falls. A prospective study. The Journal of the American Medical Association.

[B25] Sibella F, Galli M, Romei M, Montesano A, Crivellini M (2003). Biomechanical analysis of sit-to-stand movement in normal and obese subjects. Clinical Biomechanics.

[B26] Seedhom BB, Terayama K (1976). Knee forces during the activity of getting out of a chair with and without the aid of arms. Biomedical Engineering.

[B27] Ellis MI, Seedhom BB, Wright V (1984). Forces in the knee joint whilst rising from a seated position. Journal of Biomedical Engineering.

[B28] O'Meara DM, Smith RM (2006). The effects of unilateral grab rail assistance on the sit-to-stand performance of older aged adults. Human Movement Science.

[B29] O'Meara DM, Smith RM (2005). Differences between grab rail position and orientation during the assisted sit-to-stand for able-bodied older adults. Journal of Applied Biomechanics.

[B30] Csuka M, McCarty DJ (1985). Simple Method For Measurement Of Lower-Extremity Muscle Strength. American Journal of Medicine.

[B31] Guralnik JM, Simonsick EM, Ferrucci L, Glynn RJ, Berkman LF, Blazer DG, Scherr PA, Wallace RB (1994). A short physical performance battery assessing lower extremity function: association with self-reported disability and prediction of mortality and nursing home admission. Journal of Gerontology.

[B32] Jones CJ, Rikli RE, Beam WC (1999). A 30-s chair-stand test as a measure of lower body strength in community-residing older adults. Research Quarterly for Exercise and Sport.

[B33] Bohannon RW (2006). Reference values for the five-repetition sit-to-stand test: a descriptive meta-analysis of data from elders. Perceptual and Motor Skills.

[B34] Whitney SL, Wrisley DM, Marchetti GF, Gee MA, Redfern MS, Furman JM (2005). Clinical measurement of sit-to-stand performance in people with balance disorders: validity of data for the Five-Times-Sit-to-Stand Test. Physical Therapy.

[B35] Rikli RE, Jones CJ (1999). Functional fitness normative scores for community-residing older adults, ages 60-94. Journal of Aging and Physical Activity.

[B36] Kerr KM, White JA, Barr DA, Mollan RAB (1994). Standardization and definitions of the sit-stand-sit movement cycle. Gait and Posture.

